# genomicSimulation: fast R functions for stochastic simulation of breeding programs

**DOI:** 10.1093/g3journal/jkac216

**Published:** 2022-09-02

**Authors:** Kira Villiers, Eric Dinglasan, Ben J Hayes, Kai P Voss-Fels

**Affiliations:** Centre for Animal Science, Queensland Alliance for Agriculture and Food Innovation, The University of Queensland, St Lucia, 4072 QLD, Australia; Centre for Animal Science, Queensland Alliance for Agriculture and Food Innovation, The University of Queensland, St Lucia, 4072 QLD, Australia; Centre for Animal Science, Queensland Alliance for Agriculture and Food Innovation, The University of Queensland, St Lucia, 4072 QLD, Australia; Centre for Animal Science, Queensland Alliance for Agriculture and Food Innovation, The University of Queensland, St Lucia, 4072 QLD, Australia; Department of Grapevine Breeding, Hochschule Geisenheim University, Geisenheim 65366, Germany

**Keywords:** breeding program simulation, meiosis simulation, breeding program design, genomic selection, R package, C language

## Abstract

Simulation tools are key to designing and optimizing breeding programs that are multiyear, high-effort endeavors. Tools that operate on real genotypes and integrate easily with other analysis software can guide users toward crossing decisions that best balance genetic gains and genetic diversity required to maintain gains in the future. Here, we present genomicSimulation, a fast and flexible tool for the stochastic simulation of crossing and selection based on real genotypes. It is fully written in C for high execution speeds, has minimal dependencies, and is available as an R package for the integration with R’s broad range of analysis and visualization tools. Comparisons of a simulated recreation of a breeding program to a real data set demonstrate the simulated offspring from the tool correctly show key population features, such as genomic relationships and approximate linkage disequilibrium patterns. Both versions of genomicSimulation are freely available on GitHub: The R package version at https://github.com/vllrs/genomicSimulation/ and the C library version at https://github.com/vllrs/genomicSimulationC/.

## Introduction

When real breeding schemes and genetic improvement programs are high-effort, multiyear undertakings, simulation tools offer a valuable opportunity to judge feasibility or balance trade-offs in design so as to get the most out of the real programs (Li *et al.* 2012). One of the key problems in optimizing breeding scheme design is parent selection, which is highly dependent on the actual parents that are available to the breeding program (Sun *et al.* 2011; Li *et al.* 2012; Bernardo 2020).

Here, we introduce genomicSimulation, a fast, flexible R package for the stochastic simulation of breeding programs beginning with real genotype data. The package is designed to provide high-speed crossing simulation capabilities that can be easily integrated with R’s wide range of visualization and genetic analysis tools (R Core Team 2022). genomicSimulation begins with user-provided marker maps, founder genotypes, and (optional) lists of allele effect values, so can easily simulate breeding schemes on real maps and candidate founders. It simulates meiosis without mutation on alleles at discrete positions provided in the genome map. The linkage phase of the resulting genotypes is tracked. genomicSimulation works as a scripting tool, with functions for performing targeted crosses, random crosses, doubled haploids, and selfing. genomicSimulation’s inbuilt genotypic value calculator uses an additive model of marker effects. Based on the package’s range of functions, users can script their own custom selection routines in R. The package has no dependencies beyond C standard libraries. All core functionalities are written in C in order to achieve high execution speeds.

For even faster simulations, genomicSimulation’s underlying C library (in itself a fully functional simulation tool) is also available. It is distinguished from the R package only by the lack of default parameters and lack of R vector data output options.

The package was originally developed for use on self-pollinated crop species, but its flexible set of crossing operations allows it to be used more broadly in outcrossing species. genomicSimulation has full documentation, user guides, and is in ongoing development.

## Materials and methods

genomicSimulation is structured as a set of “building block” scripting functions. Users have full flexibility to intersperse requests to perform a cross and produce new offspring and restructure groups to perform selection or restructure the breeding pool.

The first step in using genomicSimulation is a call to the setup function, load.data(). Two data files are required to set up the tool: a matrix file contains the alleles of at least one founder genotype in diploid format (i.e. 2 positions per locus) at a set of discrete positions, and a linkage map to situate those positions in the model of the genome. If the phase at heterozygous positions in the founder genotypes is not known, it is randomized on load. Alleles can be any nonspace character, which allows for the package to be used on defined and known Mendelian genes as well as SNPs.

The simulation stores every simulated genotype as a sequence of characters in memory.

**Fig. 1.**
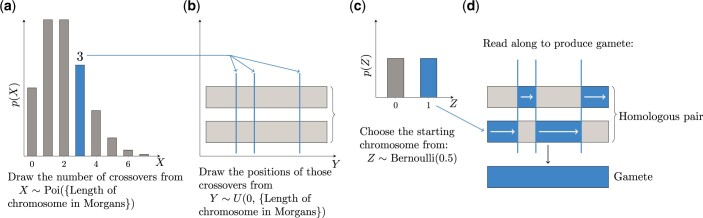
Meiosis simulation procedure in genomicSimulation uses a count-location strategy. The following steps are performed for every homologous pair of chromosomes. (A sample procedure is marked on the diagrams.) a) First, the number of crossovers is drawn from a Poisson distribution with expectation matching the length of the chromosome in Morgans. b) Next, the positions of each of those crossovers are drawn uniformly across the length of the chromosome. c) A final random draw determines which of the 2 resultant gametes is chosen. d) The gamete is created by reading along the chosen starting chromosome, swapping to the other of the pair whenever a crossover point is encountered.

To create a cross, the simulation generates a gamete independently from each of the 2 parents. No distinction is made between male and female parent at present, although the direction of the cross can be specified by the user. Karlin and Liberman’s (1979) count-location method is used to simulate meiosis (Fig. 1). First, the number of crossovers to occur in each chromosome is drawn from a Poisson distribution whose parameter is the distance in Morgans between the first and last positions tracked on that chromosome (Fig. 1a). The positions of those crossovers along the chromosome are then drawn from a uniform distribution (Fig. 1b). Finally, a random logical value (0 or 1) is drawn to choose which of the 2 possible gametes to use (Fig. 1c).

The count-location method of simulating meiosis ignores crossover interference. The specific distributions chosen add the additional assumptions that the number of crossovers is proportional to the chromosome length and that crossovers are uniformly distributed along the chromosome. This choice of distributions and assumptions is shared with the existing simulation tool Plabsoft (Maurer *et al.* 2008). Because, under this method of simulating meiosis, the number of recombinations along a segment is proportional to its length, linkage maps with distances measured in centimorgans are more appropriate than physical maps. Functionality to predetermine crossover sites or probabilities, if for example linkage disequilibrium block structure is known, will be added in future.

During setup, there is also the option to load an input file containing the numeric effects of particular SNP alleles. This enables the internal breeding value calculator to produce breeding values for individuals which are calculated using an additive model: the breeding value of a given genotype is the sum across all marker positions of each marker’s 2 alleles’ effect values (if such allele effects are loaded). If required, the breeding value can be masked to simulate phenotypes of a quantitative additive trait, e.g. by adding to the breeding value a random variable sampled from a normal distribution with a defined variance (see below). This function can be used to compare different breeding schemes with regard to rates of genetic gain for a defined trait, or to assess the genetic merit of specific crosses for the traits for which they have estimated marker effects, as a basis for crossing decisions.

The example simulation, below, makes use of genomicSimulation’s mechanisms for scripting custom selection methods to mask the breeding values calculated by the simulation tool to make a simulated phenotype. The phenotype was simulated as P=G+E, where the environmental contribution E was a normally distributed variable with mean 0 and variance $V_{e}$. Given a broad-sense heritability value for the phenotype, the variance $V_{e}$ can be calculated by rearranging:

$$H^{2}=\frac{V_{g}}{V_{g}+V_{e}}.$$

The simulated phenotypes were produced by adding a draw from the distribution E to each true breeding value. An implementation of this can be found in the example simulation. Other methods of manipulating or masking the breeding value can be similarly designed by users.

### Features

At any time after the initial setup command, more external genotypes can be loaded using the command load.more.genotypes(), and the simulation’s optional stored set of marker effects can be substituted for another with load.different.effects(). With these features, users can simulate the introduction of new genetic material into an ongoing breeding program, calculate genotypic values for multiple traits with separate sets of marker effects, and/or manually simulate environmental fluctuations across years and/or locations by using different sets of marker effects.

Simulated data can be saved to tab-separated text files or pulled into the user’s R environment as vectors.

A range of functions is available for simulating the production of offspring. These include cross.randomly(), self.n.times(), make.doubled.haploids(), and cross.all.pairs(). To carry out specific crosses or crossing plans, users can call cross.combinations() with vectors laying out the parents to cross.

Every genotype loaded or produced in genomicSimulation is allocated to a group. Mixing and separating groups allow for significant flexibility in regards to simulating multigenerational breeding pools, or having several interacting streams in the breeding program. Inbuilt (noncustom) options for group manipulation include combine.groups(), break.group.into.families(), break.group.into.halfsib.families(), and a range of functions to randomly partition a group (which can be used to demarcate male/female offspring, or to create subgroups of specific sizes for complex breeding program designs), alongside custom group manipulation using make.group().

The function select.by.gebv() performs truncation selection on breeding values calculated by the internal breeding value calculator. Custom selection methods are also possible: this involves using data from see.group.data() and some R scripting to identify the best individuals, followed by passing those individuals’ indexes to make.group() to move them into a new “selected” group. Because of the limited list of noncustom options, most selection in genomicSimulation will involve this custom selection method interface and the relevant template script from the documentation (https://vllrs.github.io/genomicSimulationC/html/templates.html).

Groups and genotypes persist until explicitly destroyed by a call to delete.groups(). Therefore, mixed-generation crossing operations are possible, and users have control over their memory resource usage. Templates for common breeding program operations are also available in the documentation.

More in-depth description of features and usage examples are provided in the R package vignette, R documentation, C library guides, and C library documentation. These can be downloaded with the package or accessed at the package GitHub links.

## Results and discussion

### Example simulation

To provide an insight into the effectiveness and flexibility of the genomicSimulation tool, a sample script for simulating a simple wheat breeding program with nonoverlapping generations is shown in Box 1. The simulation was initiated with genotype data for 50 real founder lines (Supplementary Fig. 1), and the effect values initialized with values calculated from phenotypic data for yield.

**Fig. 2.**
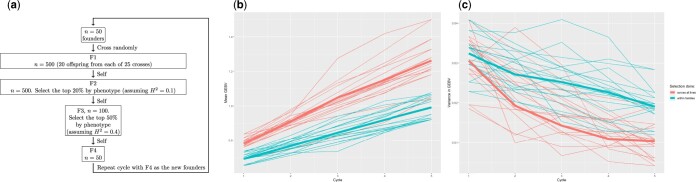
a) Diagram of each cycle of a simple breeding program plan. The program was simulated using genomicSimulation (see Box 1 for the genomicSimulation implementation). b) Mean and (c) variance in the population’s genetic breeding value are shown for each replication (thin lines) and averaged across replications (thick lines). In the first condition, the best phenotypes across the entire population in the relevant generation are selected, while in the other condition, the best phenotypes in each family are selected.

Box 1. **R script to simulate a simple breeding program using genomicSimulation. The program uses nonoverlapping cycles and selects (via the custom selection interface) after the first 2 selfing steps, with different accuracies. This version implements the “across all lines” selection condition results shown in Fig. 2, b and c; the script for the “within families” condition calls break.group.into.families() after generating the F1 crosses, then runs the rest of the script commands independently for each family group produced by the break.group command.**

g0 <- load.data(“parent-genotypes.txt,” “genetic-map.txt,” “effects.txt”)

get.top.by.phenotype <- function(group, heritability, portion) {

  info <- data.table(Index=see.group.data(group,”X”),

                     GEBV=see.group.data(group,”BV”))

  

  # simulate phenotype = genotype + environmental variation

  # using normally distributed Ve and heritability H^2 = Vg/(Vg + Ve)

  Vg <- var(info$GEBV)

  Ve <- Vg/heritability—Vg

  info$Pheno <- info$GEBV + rnorm(length(info$GEBV), mean = 0, sd = sqrt(Ve))

  

  # Select those with the top phenotype

  n <- length(info$Index) * portion

  return(make.group(info[order(info$Pheno, decreasing=TRUE),][1: n, Index]))

}

for (cyc in 1: ncycles) {

  f1 <- cross.randomly(g0, n.crosses = 25, offspring = 20)

  

  f2 <- self.n.times(f1, *n* = 1)

  f2s <- get.top.by.phenotype(f2, 0.1, 0.2)

  

  f3 <- self.n.times(f2s, *n* = 1)

  f3s <- get.top.by.phenotype(f3, 0.4, 0.5)

  

  f4 <- self.n.times(f3s, n = 1)

  f4.info <- see.group.data(f4, “B”) #result for plotting

  

  delete.group(c(g0, f1, f2, f2s, f3, f3s))

  g0 <- f4

}

The simulated breeding program was divided into cycles. The first stage of each cycle involved random crosses between founder inbred lines (using cross.randomly()) to generate new recombinants. One selfed progeny from each recombinant was “grown,” and phenotyped at 10% broad-sense heritability for the yield trait (using the breeding-value-masking method outlined above; heritability value arbitrarily chosen) to mimic selection on heterozygous potentially unreplicated material early in the breeding cycle. The top 20% of the generation by phenotype (percentage arbitrarily chosen) was selected to pass on to the next generation. One selfed progeny of each of these was “grown” and phenotyped at 40% heritability for the yield trait (number arbitrarily chosen) to mimic more accurate selection later in the breeding cycle (e.g. in early yield trials). The top 50% of that generation were selected and progressed by selfing to the last generation of the cycle. These final progeny then served as the founders of the next cycle. Figure 2a includes a visualization of the steps of the simulated breeding cycle.

In this simulation, genomicSimulation’s groups were used to represent the new population of plants grown each generation, and also to pull the selected plants out of the generation’s wider generated population.

Two conditions were tested in simulation:

1. Selection across the whole set, that is, the top 20% of all the lines in that generation is to be selected.
2. Selection within each family (the set of seeds sharing the same parent-crossed plants), that is, the top 20% of each family is to be selected.

In both scenarios, the selection intensities were simulated as described above. Each simulation condition was replicated 10 times.

The results of running the script were consistent with expectations. Figure 2b shows that the genetic value of the yield score in the population increased with each cycle, because of selection and the correlation between the phenotype and the heritable genetic trait. The condition where selection was performed across all plants showed a higher rate of gain than the condition where selection was within families, because it could select more plants from good families and therefore increase the proportion of good alleles in its population faster. Figure 2c shows the variance in genetic value scores decreased as cycles increased, as the proportion of beneficial alleles in the population and the proportion of plants with many of these alleles increased. Selecting within families kept this diversity measure higher than selecting across the whole population.

### Validation

**Fig. 3.**
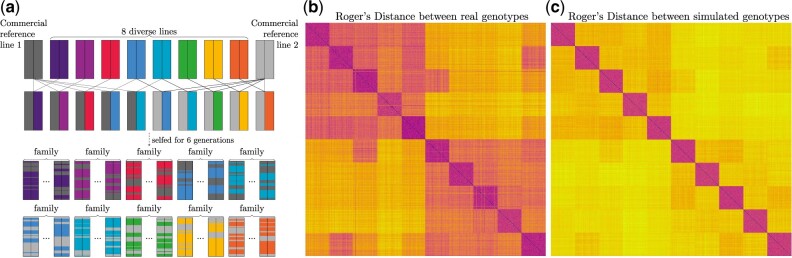
a) The crossing plan of a structured population developed by Alahmad *et al.* (2019). The matrices of Roger’s Genetic Distance between all (b) real imputed genotypes, (c) simulated genotypes, of the final-generation offspring resulting from that crossing design.

A structured durum wheat population from Alahmad *et al.* (2019) was simulated using genomicSimulation to assess the tool’s ability to recover the genetic structure found in the empirical data set. The linkage map for the markers in the empirical data set was not available, so a direct linear correlation between physical and genetic positions was assumed. The structured population was developed through the nested association mapping (NAM) design presented in Fig. 3a. This design was simulated with genomicSimulation using the cross.combinations() and self.n.times() commands to produce 10 families of 100 genotypes in the final generation.

R packages SelectionTools v. 19.4 and ComplexHeatmap v. 2.9.3 were used to produce the heatmaps of genetic (Roger’s) distance shown in Fig. 3, b and c. The population structure of 10 family relationships and 2 shared-elite-parent relationships is clearly visible in both simulated and real genotype heatmaps. The distributions of genetic distances also show the same profiles (Supplementary Fig. 2). However, the real population shows higher variation in genetic distances within families, as shown in the wider range of base colors in the heatmaps and in Supplementary Fig. 3. This may reflect a degree of assortative mating to increase variation when matings were allocated, which was not mimicked in the simulation. This may well account for the differences in level of variance observed.

**Fig. 4.**
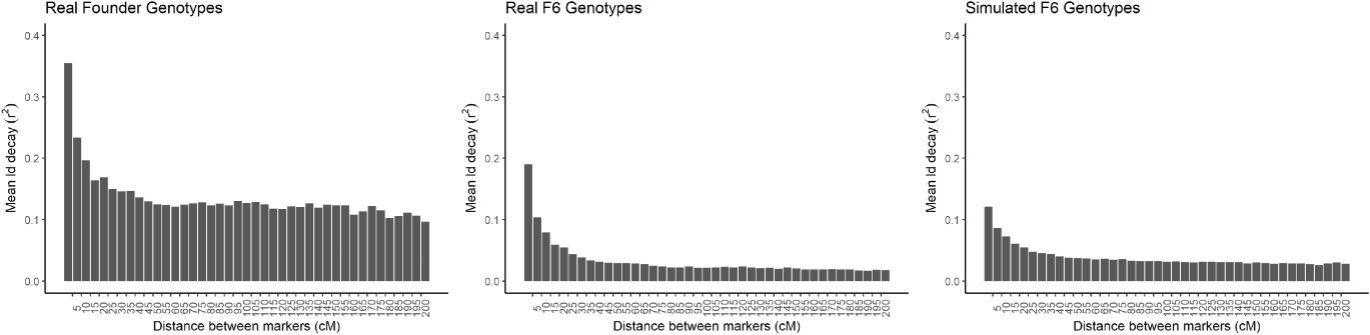
The mean LD decay ($r^{2}$) between markers in the real founding genotypes of the NAM population, the real F6 genotypes, and the simulated F6 genotypes, as a histogram on distance between markers in centiMorgans (according to the converted physical map).

Linkage disequilibrium decay of the real NAM population is slightly lower than in the simulated population (Fig. 4), probably as a result of the simple distributions used in simulating meiosis. Functionality to customize this choice of distributions, or to set crossover points and probabilities, is a development goal for genomicSimulation.

### Run speed

The tool was benchmarked against existing R packages MoBPS (Pook *et al.* 2020), AlphaSimR (Gaynor *et al.* 2020), and the Breeding Scheme Language, BSL (Yabe *et al.* 2017) (Table 1). genomicSimulation was significantly faster than most counterparts at generating large numbers of simulated genotypes. Methods to precalculate or reuse components of the breeding value calculation, to bring those compute times closer to counterparts, are under consideration. genomicSimulation offers a wider range of file-output formats than these counterpart packages, and its selection is still being developed.

**Table 1.** Average execution time (in seconds) across 6 repeats of tasks in genomicSimulation v0.2, running on a consumer-model laptop with 8 GB RAM and Intel i5-7200U CPU @ 2.50 GHz.

Mean execution time (s)	Load 50 genotypes of 5,000 SNPs	Perform 10^5^ random crosses	Get resulting breeding values	Get resulting genotypes
genomicSimulationC	0.66	4.26	70.13	67.99
genomicSimulation (in RStudio)	0.97	2.05	74.60	108.18
MoBPS (in RStudio)	0.74	193.99	0.24	No equivalent
AlphaSimR (in RStudio)	0.25	15.47	3.12	102.29
BreedingSchemeLanguage (in RStudio)	1.19	*	*	*

The benchmarks are compared to the times taken to perform comparable tasks in simulation tools MoBPS (Pook *et al.* 2020), AlphaSimR (Gaynor *et al.* 2020), and Breeding Scheme Language (Yabe *et al.* 2017). The tasks benchmarked are: (1) loading 50 genotypes of 5,000 SNPs, (2) perform 100,000 random crosses between those genotypes with one progeny per cross, (3) calculate then save the breeding values of the 10^5^ genotypes from task 2 to an R dataframe (except for genomicSimulationC, which rather saves them to a file), and (4) save the 10^5^ genotypes from task 2 to a file. Note that the R version of genomicSimulationalso shares the C library’s functionality for saving simulated data to files rather than to R dataframes. The time taken to save output to files is comparable across R and C versions. Cells marked with an asterisk (*) mark tasks that could not be benchmarked due to memory limitations on the testing machine.

### Summary

Simulation is a valuable tool to investigate the choices needed to carry out a breeding program. Various simulation tools in this area exist. Some, such as QU-GENE (Podlich and Cooper 1998) and ADAM-Plant (Liu *et al.* 2019), are standalone programs, but an increasing number are R packages. These include Plabsoft (Maurer *et al.* 2008), BSL (Yabe *et al.* 2017), MoBPS (Pook *et al.* 2020), and AlphaSimR (Gaynor *et al.* 2020). genomicSimulation joins these ranks as a simple, flexible, freely available tool designed for simulation and decision-making relating to real genome maps and potential founders at high speeds. It is available as a standalone command-line tool or as a set of R scripting commands.

R’s popularity in biological data analysis means it houses a range of cutting-edge genetic analysis and genomic selection packages with which users may already be familiar. Developing a breeding program simulation in base R, however, requires significant effort and planning, and may produce a slow-running tool. genomicSimulation provides an engine to carry out simulations according to breeding programs that can be scripted in R. At present, it offers an additive internal breeding value calculator, although this offering will be expanded to include nonadditive genetic effects. It is expected that users script more complex genetic evaluation methods themselves, taking advantage of the available templates/guides available in the documentation (https://vllrs.github.io/genomicSimulationC/html/templates.html), for example, to simulate a phenotype (as in the example in this article), or recalculate and reload SNP effect values.

Its approachable syntax, easy interoperability with other R libraries, and automatically fast execution (thanks to the underlying C library implementation) make it a useful tool to simulate, test, and compare breeding strategies. genomicSimulation should be straightforward to install thanks to its lack of dependencies beyond C standard libraries. Documentation and use guides are available. Development is ongoing.

## Supplementary Material

jkac216_Supplemental_Figures_Supplementary_Material_1

jkac216_Supplementary_Material_2

jkac216_Supplementary_Material_3

jkac216_Supplementary_Material_4

jkac216_Supplementary_Material_5

jkac216_Supplementary_Material_6

jkac216_Supplementary_Material_7

jkac216_Supplementary_Material_8

## Data Availability

Code and installations of both versions of genomicSimulation are freely available on GitHub: The R package version at https://github.com/vllrs/genomicSimulation/ and the C library version at https://github.com/vllrs/genomicSimulationC/. The data underlying this article are available in the article and in its online supplementary material. Code used to run the Example Simulation is provided in Supplementary Material 2. The input data for the Example Simulation is available in Supplementary Materials 3 through 5 (respectively, the founder genotypes, the map, and the marker effects). Code used to run the Validation simulation is provided in Supplementary Material 6. The input data for this simulation are available in Supplementary Materials 7 and 8 (respectively, the founder genotypes and the map). Supplemental material is available at G3 online.
